# Added Diagnostic Value of Stress CT Perfusion to Coronary CT Angiography Across Increasing Coronary Calcium Burden

**DOI:** 10.3390/jimaging12100463

**Published:** 2026-09-22

**Authors:** Marco Fogante, Enrico Paolini, Paolo Esposto Pirani, Nicolò Schicchi

**Affiliations:** 1 Maternal-Child, Senological, Cardiological Radiology and Outpatient Ultrasound, Department of Radiological Sciences, University Hospital of Marche, 60126 Ancona, Italy; paolo.espostopirani@ospedaliriuniti.marche.it (P.E.P.); nicolo.schicchi@ospedaliriuniti.marche.it (N.S.); 2 Cardiology and Intensive Cardiac Care Unit, Cardiovascular Department, University Hospital of Marche, 60126 Ancona, Italy; enrico.paolini@ospedaliriuniti.marche.it

**Keywords:** coronary CT angiography, CT myocardial perfusion, coronary artery calcium, stress CT perfusion, diagnostic accuracy, coronary artery disease

## Abstract

Coronary CT angiography (CCTA) may show reduced specificity in patients with extensive coronary artery calcification (CAC). We evaluated the diagnostic performance of CCTA alone versus integrated CCTA–stress myocardial CT perfusion (CTP) across different CAC burdens. In this retrospective single-center study, 102 symptomatic patients with suspected coronary artery disease underwent CCTA with stress-only dynamic CTP and were stratified into CAC 0–400 (n = 38), 401–1000 (n = 43), and >1000 (n = 21). Stress-induced hypoperfusion was defined using a prespecified relative myocardial blood flow ratio ≤ 0.85. The primary diagnostic-accuracy analysis included 68 patients with an invasive reference standard (ICA/QCA ± FFR); FFR ≤ 0.80 was used when available, whereas QCA stenosis ≥ 50% defined reference-positive disease otherwise. The remaining 34 patients had complete event-free 12-month clinical follow-up and were included exclusively in a supportive exploratory analysis. In the primary cohort, CCTA alone showed a sensitivity of 89.8%, specificity of 36.8%, and accuracy of 75.0%, compared with 87.8%, 84.2%, and 86.8%, respectively, for integrated CCTA–CTP. The integrated strategy increased specificity by 47.4 percentage points (95% CI, 24.9–69.8) and accuracy by 11.8 percentage points (95% CI, 3.1–20.4), with a significant paired difference (McNemar *p* = 0.021) and an NRI of 0.45 (95% CI, 0.22–0.68). In the supportive exploratory full-cohort analysis, integrated CCTA–CTP showed numerically higher accuracy across all CAC strata; however, CAC-stratified comparisons were not individually significant and these findings should be considered descriptive. Mean total radiation dose was 7.0 ± 1.4 mSv. Integrated stress CTP was associated with higher specificity and diagnostic accuracy than CCTA alone, primarily by reducing false-positive anatomical classifications.

## 1. Introduction

Coronary computed tomography angiography (CCTA) is widely established as a first-line non-invasive imaging modality for the evaluation of patients with suspected coronary artery disease (CAD), owing to its high sensitivity, excellent negative predictive value, and well-established prognostic value [1,2]. Current international guidelines support its use as an initial diagnostic test in appropriately selected symptomatic patients, confirming its central role in contemporary CAD assessment [3].

Despite these advantages, the diagnostic performance of CCTA may be impaired by extensive coronary calcification. In patients with high coronary artery calcium (CAC) burden, blooming and partial-volume artifacts can lead to overestimation of stenosis severity, reducing specificity and overall diagnostic accuracy while sensitivity generally remains preserved [4,5]. Consequently, anatomical assessment may become increasingly challenging in heavily calcified coronary arteries, with a higher rate of false-positive findings and potentially unnecessary downstream testing [6].

Functional assessment provides complementary information beyond anatomical stenosis severity. Stress myocardial CT perfusion (CTP) enables evaluation of myocardial perfusion within the same imaging session as CCTA and has shown good diagnostic performance compared with invasive and non-invasive reference standards, including fractional flow reserve (FFR) and established functional imaging techniques [7,8,9,10,11,12]. The integration of CCTA and stress CTP may therefore help reduce false-positive anatomical classifications, particularly when coronary calcification limits reliable lumen assessment.

Previous studies, including CORE320 and more recent investigations using third-generation dual-source CT, have already demonstrated the incremental diagnostic value of CTP when combined with CCTA [13,14]. Despite the established value of CCTA, diagnostic confidence decreases in the presence of moderate-to-severe coronary calcification because blooming artifacts and limited lumen visualization may increase false-positive stenosis assessment. In this setting, an additional functional evaluation may help distinguish anatomically suspicious lesions from those associated with relevant downstream perfusion abnormalities. However, the role of a stress-only dynamic CTP approach based on relative myocardial blood flow (MBF) across different CAC burdens has not been fully characterized.

The aim of this study was therefore to compare the diagnostic performance of CCTA alone with that of integrated CCTA–stress CTP in patients with suspected CAD across increasing CAC burden, using both per-patient and per-vessel analyses and evaluating the incremental reclassification provided by the combined approach.

## 2. Materials and Methods

### 2.1. Study Design and Population

This retrospective single-center observational study was conducted at a tertiary referral institution. The study was approved by the Ethics Committee of University Hospital of Marche (ID: 4191; date of approval: 19 June 2025). All patients had provided written informed consent for the clinically indicated CT examination and for the use of anonymized data for research purposes.

Consecutive symptomatic patients undergoing clinically indicated CCTA with integrated stress CTP for suspected CAD between January 2022 and December 2024 were identified from the institutional database. At our institution, stress CTP was selectively added when further functional assessment was considered clinically useful, particularly in patients with intermediate-to-high clinical likelihood of CAD, moderate or equivocal stenosis, extensive coronary calcification limiting anatomical assessment, or discordance between symptoms and CCTA findings. Thus, integrated CCTA–CTP was not routinely performed in all patients referred for CCTA.

Inclusion criteria were availability of CAC scoring, CCTA, and stress CTP acquired during the same examination, together with either ICA/QCA ± FFR or complete 12-month clinical follow-up. Patients with prior coronary revascularization or known myocardial scar were excluded. Of 156 screened patients, 54 were excluded because of prior revascularization (n = 25), previous myocardial infarction with known scar (n = 9), absence of ICA or complete follow-up (n = 13), or incomplete clinical data (n = 7), leaving 102 patients for analysis.

Among these, 68 patients underwent ICA/QCA, including 22 with FFR, and constituted the primary diagnostic-accuracy cohort. The remaining 34 patients had complete 12-month clinical follow-up and were included only in a supportive exploratory patient-level analysis, with exclusion from primary and per-vessel analyses (Figure 1).

Complete follow-up required clinical information covering the entire 12-month period. For the supportive exploratory full-cohort analysis, patients without ICA were classified as reference-positive if at least one qualifying coronary event occurred during follow-up, defined as myocardial infarction, hospitalization for unstable angina, or coronary revascularization. Patients with complete 12-month follow-up and no qualifying event were classified as reference-negative. This event-based classification was used exclusively for the supportive exploratory analysis and was not considered equivalent to an invasive reference standard.

### 2.2. CT Acquisition Protocol

All examinations were performed on a third-generation dual-source CT scanner (SOMATOM Force, Siemens Healthineers, Erlangen, Germany). Patients abstained from caffeine for at least 24 h before imaging. The protocol included non-contrast ECG-triggered CAC scoring, ECG-gated CCTA, and stress-only dynamic myocardial CTP within the same session.

For CCTA, 40 mL of iodinated contrast medium (370 mg I/mL) was injected at 5 mL/s, followed by a 40-mL saline flush. Tube voltage was automatically selected between 90 and 120 kV with automated tube-current modulation. Acquisition was performed with 96 × 0.6-mm collimation and a 250-ms gantry rotation time. Images were reconstructed at 0.6-mm slice thickness with a 0.4-mm increment using a medium-smooth vascular kernel and ADMIRE level 3.

Pharmacological stress was induced with intravenous regadenoson (0.4 mg). Dynamic CTP was initiated 60–90 s after administration, using 60 mL of iodinated contrast at 6 mL/s followed by a 40-mL saline flush. Acquisition was performed in shuttle mode at 80 kV and approximately 300 mAs/rotation, with 96 × 0.6-mm collimation and approximately 10.5-cm z-axis coverage. Images were acquired over approximately 30 s at ~1.5-s temporal sampling, generating 15–20 perfusion phases, and reconstructed at 3-mm slice thickness with a 2-mm increment using iterative reconstruction.

### 2.3. Radiation Dose

Radiation exposure was recorded separately for CAC scoring, CCTA, and stress CTP using scanner-reported dose-length product (DLP) and CTDIvol values. Effective dose was estimated from DLP using a cardiac-specific conversion factor of 0.015 mSv·mGy^−1^·cm^−1^, and total effective dose was calculated as the sum of all examination components.

### 2.4. Image Interpretation and Reference Standard

Images were analyzed using dedicated software (syngo.via, VB10A; Siemens Healthineers, Forchheim and Erlangen, Germany). CAC was quantified using the Agatston method and categorized as 0–400, 401–1000, or >1000. CCTA and stress CTP were independently interpreted by two cardiovascular radiologists with >10 years of experience, blinded to clinical data, ICA/FFR findings, and each other’s assessments; disagreements were resolved by consensus with a third senior reader.

Stress CTP hypoperfusion was defined as a ≥15% reduction in myocardial blood flow relative to visually normal myocardium within the same examination (MBF ratio ≤0.85). Quantitative MBF maps were generated on syngo.via, and the MBF ratio was calculated as MBF in the suspected hypoperfused region divided by mean MBF in artifact-free, visually normal reference myocardium. Segments supplied by vessels with potentially significant upstream stenosis and areas affected by motion, beam-hardening, or partial-volume artifacts were avoided whenever possible. The ≤0.85 threshold was prespecified from previous dynamic stress CTP studies [15,16]. As an exploratory internal validation, ROC analysis was performed in the 68-patient invasive-reference cohort using the lowest relative MBF ratio per patient, and the optimal cutoff was determined using the Youden index.

CCTA-only interpretation was completed and recorded before review of the stress CTP datasets. Once finalized, the anatomical CCTA classification was locked and could not be modified after perfusion assessment. CTP was then reviewed and integrated with the previously recorded CCTA findings.

For the primary analysis, CCTA positivity was defined as visually estimated stenosis ≥50% in at least one coronary segment. Integrated CCTA–CTP positivity required a ≥50% CCTA stenosis associated with downstream stress-induced hypoperfusion. Because invasive physiological assessment was available only in a subset of patients, the integrated classification was not considered synonymous with hemodynamically significant CAD.

ICA/QCA, with FFR when available, served as the invasive reference standard. In vessels without FFR, QCA stenosis ≥50% was considered reference-positive and <50% reference-negative. When FFR was available, FFR ≤0.80 defined reference-positive and FFR > 0.80 reference-negative status. At the patient level, reference-positive CAD was defined by the presence of at least one major coronary vessel fulfilling these criteria. Accordingly, the primary endpoint was termed “reference-positive CAD” to reflect the hybrid invasive reference standard.

In equivocal cases in which ischemia could not be confidently distinguished from unrecognized scar, additional tissue-characterization imaging, such as CMR with late gadolinium enhancement or delayed iodine-enhancement CT, was considered appropriate.

### 2.5. Statistical Analysis

Continuous variables are reported as mean ± standard deviation or median with interquartile range, as appropriate, and categorical variables as counts and percentages. Comparisons across CAC categories were performed using one-way ANOVA or the Kruskal–Wallis test for continuous variables and the χ^2^ test or Fisher’s exact test for categorical variables, as appropriate.

The primary diagnostic-accuracy analysis included the 68 patients with an invasive reference standard (ICA/QCA ± FFR). Sensitivity, specificity, PPV, NPV, and overall accuracy were calculated for CCTA alone and integrated CCTA–CTP at the per-patient and per-vessel levels using a ≥50% CCTA stenosis threshold. All estimates were referenced to the hybrid invasive definition of reference-positive CAD and were not interpreted as direct measures of hemodynamically significant CAD.

Diagnostic-performance estimates are reported with two-sided 95% CIs. Per-patient CIs were calculated using the exact Clopper–Pearson method. Paired differences in sensitivity, specificity, and accuracy between CCTA alone and integrated CCTA–CTP were also reported with 95% CIs.

Per-vessel analyses were restricted to the invasive-reference cohort and accounted for within-patient correlation using patient-level cluster bootstrap resampling. Patients were resampled with replacement, retaining all evaluable vessels from each selected patient. Cluster-adjusted 95% CIs and paired differences were derived from 2000 bootstrap replications.

A secondary sensitivity analysis used a ≥70% CCTA stenosis threshold while maintaining the same invasive reference-standard definition. The same confidence-interval and clustering procedures were applied.

At the per-patient level, paired comparisons were performed using the exact McNemar test. Category-based NRI was calculated using two diagnostic categories (test-positive and test-negative). For the primary ≥50% analysis, CCTA positivity required stenosis ≥50%, whereas integrated CCTA–CTP positivity required a ≥50% stenosis with downstream hypoperfusion (relative MBF ratio ≤0.85). Event and non-event NRI components were calculated, with 95% CIs estimated using stratified patient-level bootstrap resampling within reference-positive and reference-negative groups.

A supportive exploratory patient-level analysis was also performed in the full cohort of 102 patients, including the 34 patients with clinical follow-up only, who were classified according to the prespecified event-based rule described above. All full-cohort estimates were considered secondary to the primary invasive-reference analysis. Because event-free clinical follow-up cannot exclude anatomically obstructive CAD or inducible myocardial ischemia, this approach may result in disease misclassification and overestimate specificity. Accordingly, the full-cohort analysis was not used to derive the primary diagnostic-accuracy conclusions.

CAC-stratified analyses were considered exploratory. No formal interaction test between CAC category and imaging strategy was performed because of limited subgroup size and the small number of reference-negative patients; between-stratum differences were therefore interpreted descriptively.

Inter-observer agreement was assessed using Cohen’s kappa for categorical variables and intraclass correlation coefficients for continuous perfusion parameters. All tests were two-sided, with *p* < 0.05 considered statistically significant.

## 3. Results

### 3.1. Study Population

A total of 102 patients were included and stratified according to CAC burden: CAC 0–400 (n = 38), CAC 401–1000 (n = 43), and CAC > 1000 (n = 21).

Stress-induced myocardial hypoperfusion was detected in 48 patients (47.1%), with a median of 2 involved segments per patient (IQR, 1–3). The LAD territory was most frequently affected (27 patients, 56.3%), followed by the RCA (18 patients, 37.5%) and LCx (14 patients, 29.2%); multiterritorial hypoperfusion was observed in 15 patients (31.3%).

Exploratory ROC analysis identified an optimal relative MBF ratio cutoff of 0.84 by the Youden index, closely matching the prespecified threshold of 0.85.

Among the 68 patients with an invasive reference standard, 49 (72.1%) were reference-positive and 19 (27.9%) reference-negative. FFR was performed in 22 patients and was available for 36 of the 204 evaluated major coronary vessels (17.6%); the remaining 168 vessels (82.4%) were classified according to QCA alone. None of the 34 patients with clinical follow-up only experienced a qualifying coronary event during the 12-month follow-up (0/34, 0%); therefore, all 34 were classified as reference-negative exclusively for the supportive exploratory full-cohort analysis. Baseline characteristics are reported in Table 1.

### 3.2. Radiation Dose

Mean CTDIvol and DLP were 2.1 ± 0.6 mGy and 24 ± 7 mGy·cm for CAC scoring, 10.2 ± 2.7 mGy and 123 ± 35 mGy·cm for CCTA, and 30.5 ± 7.0 mGy and 320 ± 73 mGy·cm for stress CTP, respectively. Using a cardiac-specific conversion factor of 0.015 mSv·mGy^−1^·cm^−1^, the mean effective dose was 2.2 ± 0.6 mSv for CAC scoring plus CCTA, 4.8 ± 1.1 mSv for stress CTP, and 7.0 ± 1.4 mSv for the complete integrated protocol.

### 3.3. Primary Per-Patient Diagnostic Performance

In the 68-patient invasive-reference cohort, 49 patients were reference-positive and 19 reference-negative. Because FFR was available only in a subset, diagnostic performance refers to the hybrid invasive reference standard rather than specifically to hemodynamically significant CAD.

CCTA alone showed a sensitivity of 89.8% (95% CI, 77.8–96.6%), specificity of 36.8% (95% CI, 16.3–61.6%), PPV of 78.6% (95% CI, 65.6–88.4%), NPV of 58.3% (95% CI, 27.7–84.8%), and accuracy of 75.0% (95% CI, 63.0–84.7%). Integrated CCTA–CTP showed corresponding values of 87.8% (95% CI, 75.2–95.4%), 84.2% (95% CI, 60.4–96.6%), 93.5% (95% CI, 82.1–98.6%), 72.7% (95% CI, 49.8–89.3%), and 86.8% (95% CI, 76.4–93.8%), respectively.

Thus, integrated CCTA–CTP showed higher specificity and overall accuracy, with similar sensitivity. CAC-stratified results are reported in Table 2.

### 3.4. Supportive Exploratory Full-Cohort Analysis

In the supportive exploratory full-cohort analysis of 102 patients, which included 34 patients classified according to event-free 12-month clinical follow-up rather than an invasive reference standard, integrated CCTA–CTP showed numerically higher diagnostic accuracy across all CAC strata, increasing from 81.6% to 92.1% for CAC 0–400, from 79.1% to 88.4% for CAC 401–1000, and from 71.4% to 85.7% for CAC > 1000. However, none of the CAC-stratified paired comparisons reached statistical significance; these findings should therefore be considered descriptive and supportive rather than evidence of a CAC-dependent effect.

Uncertainty was greatest in the CAC > 1000 subgroup, where only five patients were reference-negative. In this group, specificity increased from 40.0% (2/5; 95% CI, 5.3–85.3%) to 80.0% (4/5; 95% CI, 28.4–99.5%), corresponding to only two additional correctly classified patients.

Detailed results are reported in Table 3.

### 3.5. Primary Per-Vessel Diagnostic Performance

The primary per-vessel analysis included 204 major coronary vessels from the 68 patients with an invasive reference standard, of which 79 (38.7%) were reference-positive. Within-patient correlation was accounted for using patient-level cluster bootstrap analysis. FFR was available for 36/204 vessels (17.6%) in 22 patients, whereas the remaining 168/204 vessels (82.4%) were classified according to QCA alone.

CCTA alone maintained high sensitivity across CAC strata but showed lower specificity and diagnostic accuracy with increasing CAC burden. Integrated CCTA–CTP showed higher specificity and accuracy, with a modest reduction in sensitivity. Given the limited subgroup sizes, CAC-stratified vessel-level findings were considered exploratory.

Overall, sensitivity, specificity, and diagnostic accuracy were 88.6%, 51.2%, and 65.7% for CCTA alone versus 82.3%, 84.0%, and 83.3% for integrated CCTA–CTP, respectively. Cluster-adjusted 95% CIs are reported in Table 4. Figure 2 summarizes the complementary diagnostic roles of CCTA and stress CTP across increasing CAC burden. Figure 3, Figure 4 and Figure 5 show representative cases across the CAC spectrum, illustrating concordant and discordant anatomical and perfusion findings.

### 3.6. Inter-Observer Agreement

Inter-observer agreement was high for both imaging strategies. For CCTA-based classification using the ≥50% stenosis threshold, agreement was substantial at the per-patient (κ = 0.82) and per-vessel (κ = 0.78) levels. Agreement for integrated CCTA–CTP interpretation was similarly high (κ = 0.85 and κ = 0.80, respectively). Quantitative stress CTP analysis also showed good reproducibility, with ICC values of 0.87 for absolute MBF and 0.90 for the relative MBF ratio.

### 3.7. Sensitivity Analysis Using a ≥70% CCTA Stenosis Threshold

A sensitivity analysis using a more stringent ≥70% CCTA stenosis threshold yielded findings consistent with the primary analysis. In the 68-patient invasive-reference cohort, CCTA alone showed a sensitivity of 77.6% (95% CI, 63.4–88.2%), specificity of 78.9% (95% CI, 54.4–93.9%), and diagnostic accuracy of 77.9% (95% CI, 66.2–87.1%). The corresponding values for integrated CCTA–CTP were 75.5% (95% CI, 61.1–86.7%), 84.2% (95% CI, 60.4–96.6%), and 77.9% (95% CI, 66.2–87.1%), respectively. CAC-stratified results are reported in Table 5.

At the per-vessel level, CCTA alone showed a sensitivity of 77.2% (95% CI, 66.4–85.9%), specificity of 74.4% (95% CI, 65.8–81.8%), and accuracy of 75.5% (95% CI, 69.0–81.2%). Integrated CCTA–CTP showed a sensitivity of 72.2% (95% CI, 60.9–81.7%), specificity of 88.8% (95% CI, 81.9–93.7%), and accuracy of 82.4% (95% CI, 76.4–87.3%). The CAC-stratified estimates remained supportive and exploratory and are presented in Table 6.

In the supportive exploratory full cohort of 102 patients, diagnostic accuracy was 82.4% (95% CI, 73.6–89.2%) for CCTA alone and 85.3% (95% CI, 76.9–91.5%) for integrated CCTA–CTP. These full-cohort estimates were considered secondary and supportive because 34 patients lacked an invasive reference standard. The main numerical effect of adding stress CTP at the ≥70% threshold remained an increase in specificity, whereas sensitivity was slightly reduced (Table 7).

### 3.8. Comparative Diagnostic Performance Analysis

In the primary invasive-reference cohort of 68 patients, integrated CCTA–CTP significantly modified diagnostic classification compared with CCTA alone. Among 10 discordant cases, 9 false-positive CCTA findings were correctly reclassified as true-negative, whereas 1 true-positive case was reclassified as false-negative (exact McNemar *p* = 0.021).

Compared with CCTA alone, integrated CCTA–CTP was associated with an absolute change in sensitivity of −2.0 percentage points (95% CI, −6.0 to 1.9), an increase in specificity of 47.4 percentage points (95% CI, 24.9–69.8), and an increase in overall diagnostic accuracy of 11.8 percentage points (95% CI, 3.1–20.4). The overall NRI was 0.45 (95% CI, 0.22–0.68), mainly driven by improved classification of reference-negative patients and reduction in false-positive CCTA findings (non-event NRI 0.474; event NRI −0.020).

In the supportive exploratory full-cohort analysis of 102 patients, integrated CCTA–CTP corrected 12 false-positive classifications while introducing 1 additional false-negative case (exact McNemar *p* = 0.003). The overall NRI was 0.21 (95% CI, 0.09–0.32). These findings were considered secondary and supportive because the 34 patients without ICA were classified as reference-negative on the basis of event-free clinical follow-up rather than an invasive reference standard; consequently, these estimates may be affected by overestimation of specificity.

## 4. Discussion

In this retrospective single-center study, we evaluated whether the addition of stress myocardial CTP could improve diagnostic classification when anatomical CCTA assessment is challenged by coronary calcification. Calcified plaques are a well-recognized limitation of CCTA because blooming and partial-volume artifacts may impair lumen visualization, overestimate stenosis severity, and increase false-positive findings [4]. This limitation becomes progressively more relevant with increasing CAC burden, particularly above 400 Agatston units [6,13,14].

In the primary cohort of 68 patients with an invasive reference standard, integrated CCTA–stress CTP showed higher specificity and overall diagnostic accuracy than CCTA alone, while maintaining similar sensitivity. The main effect of adding perfusion information was a reduction in false-positive anatomical classifications. Importantly, these findings should be interpreted against the hybrid invasive reference standard used in this study. Because FFR was available only in a subset of patients, the observed improvement cannot be considered direct evidence of better identification of hemodynamically significant CAD, but rather improved classification relative to ICA/QCA ± FFR.

The diagnostic challenge posed by coronary calcification persists despite contemporary CT technology. Third-generation dual-source CT, automatic exposure modulation, and iterative reconstruction can improve image quality and partially reduce calcium-related artifacts, but extensive calcification may still compromise anatomical stenosis assessment [17]. More recently, photon-counting CT has further improved spatial resolution and reduced blooming artifacts in heavily calcified coronary arteries [18]. Nevertheless, these techniques remain primarily anatomical and do not directly assess downstream myocardial perfusion. Stress CTP may therefore provide complementary functional information within the same CT examination, allowing combined assessment of coronary anatomy and myocardial perfusion [19,20].

Our findings are consistent with previous studies evaluating combined anatomical and functional CT strategies. CORE320 demonstrated incremental diagnostic value of myocardial CTP over CCTA across increasing CAC burden [13], while Ladeiras-Lopes et al. showed improved diagnostic performance of integrated CCTA–CTP in patients with high coronary calcium, reporting an AUC of 0.84 in patients with CAC > 400 [6]. Accordingly, the novelty of the present study does not lie in demonstrating a general incremental benefit of CTP over CCTA, but in evaluating a stress-only relative-MBF strategy across three CAC categories, including a dedicated CAC > 1000 subgroup, together with paired diagnostic reclassification analysis.

Numerically higher diagnostic performance with integrated CCTA–CTP was observed across CAC strata at both the per-patient and per-vessel levels. However, the individual CAC-stratified comparisons were not statistically significant, and no formal interaction between CAC burden and imaging strategy was demonstrated. These subgroup findings should therefore be regarded as exploratory and hypothesis-generating rather than evidence that the incremental benefit of CTP increases with CAC severity. The relatively small number of patients, particularly in the CAC > 1000 group, also limits statistical power and leaves open the possibility of Type II error.

The sensitivity analysis using a more stringent ≥70% CCTA stenosis threshold provided additional support for the main findings. As expected, increasing the anatomical threshold improved the specificity of CCTA alone compared with the primary ≥50% analysis. Nevertheless, integrated CCTA–CTP continued to show higher specificity and overall diagnostic accuracy at both the per-patient and per-vessel levels, with a modest reduction in sensitivity. This suggests that the complementary contribution of stress perfusion was not restricted to the conventional ≥50% CCTA threshold.

An important methodological feature of the present study was the use of a stress-only dynamic CTP protocol combined with a relative MBF ratio. Stress-only strategies may simplify the examination and reduce radiation exposure by avoiding an additional rest acquisition. Relative MBF parameters may also reduce variability related to patient-specific hemodynamics and acquisition conditions. The prespecified MBF ratio threshold of ≤0.85 was derived from previous studies [15,16], and exploratory ROC analysis identified a closely corresponding optimal cutoff in the present cohort. Previous investigations have similarly supported the use of relative perfusion parameters for identifying stress-induced myocardial hypoperfusion [21,22,23].

Reproducibility represents an additional consideration for the clinical application of myocardial perfusion CT. In the present study, interpretation was performed independently by two experienced cardiovascular radiologists, with adjudication by a third reader when necessary. Inter-observer agreement was substantial for CCTA and integrated CCTA–CTP interpretation, while quantitative perfusion measurements also showed good reproducibility. These findings support the robustness of the approach when standardized acquisition protocols, dedicated post-processing software, and predefined interpretation criteria are used.

Radiation exposure remains an important consideration in comprehensive cardiac CT imaging. The stress-only strategy, combined with third-generation dual-source CT technology, resulted in a mean total effective dose of approximately 7 mSv for the complete integrated examination. This exposure is within the range reported for contemporary optimized cardiac CT protocols and represents one of the reasons for avoiding an additional rest perfusion acquisition [9].

From a clinical perspective, the observed reduction in false-positive classifications suggests that stress CTP may be useful when coronary calcification limits reliable anatomical CCTA interpretation. However, the present study does not identify a specific CAC threshold above which CTP should routinely be added, nor was it designed to evaluate downstream ICA use, changes in patient management, or clinical outcomes. The present findings should therefore support the complementary role of perfusion assessment rather than define a CAC-guided diagnostic pathway.

This study has several limitations. First, its retrospective single-center design and the clinically selected use of integrated CCTA–CTP may limit generalizability. Patients referred for stress CTP likely had a higher pre-test likelihood of CAD and greater diagnostic complexity than an unselected CCTA population, potentially affecting disease prevalence and the baseline specificity of CCTA. Exclusion of patients without either ICA or complete 12-month follow-up may have introduced additional selection bias. Second, the sample size within individual CAC categories was limited, particularly in the CAC > 1000 subgroup. Consequently, CAC-stratified estimates have wide uncertainty, the study was not powered to demonstrate an interaction between CAC burden and the incremental effect of CTP, and Type II error cannot be excluded. Third, ICA was not available in all patients. The primary diagnostic-accuracy analysis was therefore restricted to the patients undergoing ICA/QCA, with FFR when available, while the patients with clinical follow-up only were included exclusively in a supportive exploratory patient-level analysis. Event-free follow-up, however, cannot exclude anatomically obstructive CAD or inducible myocardial ischemia and may consequently have resulted in disease misclassification and overestimation of specificity. Accordingly, the full-cohort estimates should be interpreted as supportive rather than as equivalent to the primary invasive-reference analysis. Furthermore, clinically driven referral to ICA introduces potential referral and partial-verification bias. FFR was not systematically performed; therefore, the present study cannot specifically establish diagnostic performance for hemodynamically significant CAD. Fourth, CAC burden was characterized by the Agatston score without systematic assessment of calcium morphology or distribution. Focal versus diffuse and eccentric versus concentric calcification may influence blooming artifacts and lumen interpretability independently of total calcium burden and may therefore modify the incremental contribution of CTP. Future studies should incorporate both quantitative and morphological characterization of coronary calcification. Finally, the stress-only protocol does not allow direct rest–stress comparison and may limit differentiation between inducible hypoperfusion and previously unrecognized fixed myocardial scar. This potential confounding effect was reduced by excluding patients with known previous myocardial infarction or myocardial scar; however, additional tissue-characterization imaging may be required in equivocal cases.

## 5. Conclusions

In patients with suspected CAD and an invasive reference standard, integrated CCTA–stress CTP showed higher specificity and overall diagnostic accuracy than CCTA alone, mainly by reducing false-positive anatomical classifications. Although numerically favorable findings were observed across CAC strata, these subgroup results remain exploratory and do not demonstrate a CAC-dependent increase in benefit.

Stress CTP may therefore provide useful complementary perfusion information when coronary calcification limits anatomical CCTA assessment. However, these findings do not establish a CAC-based diagnostic pathway or support specific recommendations regarding downstream ICA, management decisions, or clinical outcomes.

## Figures and Tables

**Figure 1:**
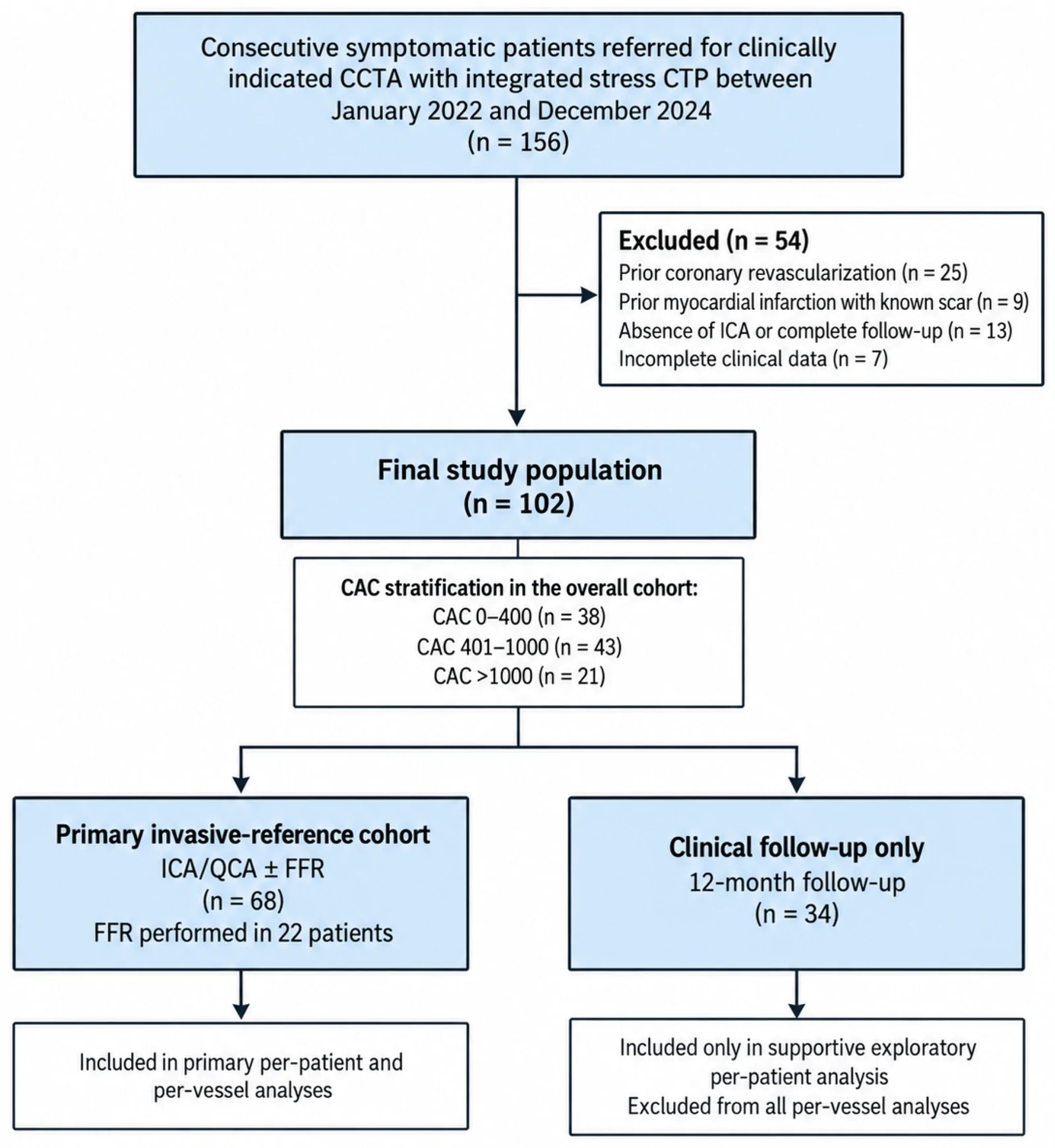
Flow-chart illustrating patient selection and exclusion criteria. **Abbreviations**—CCTA, coronary computed tomography angiography; CTP, computed tomography perfusion; CAC, coronary artery calcium; ICA, invasive coronary angiography; QCA, quantitative coronary angiography; FFR, fractional flow reserve.

**Figure 2:**
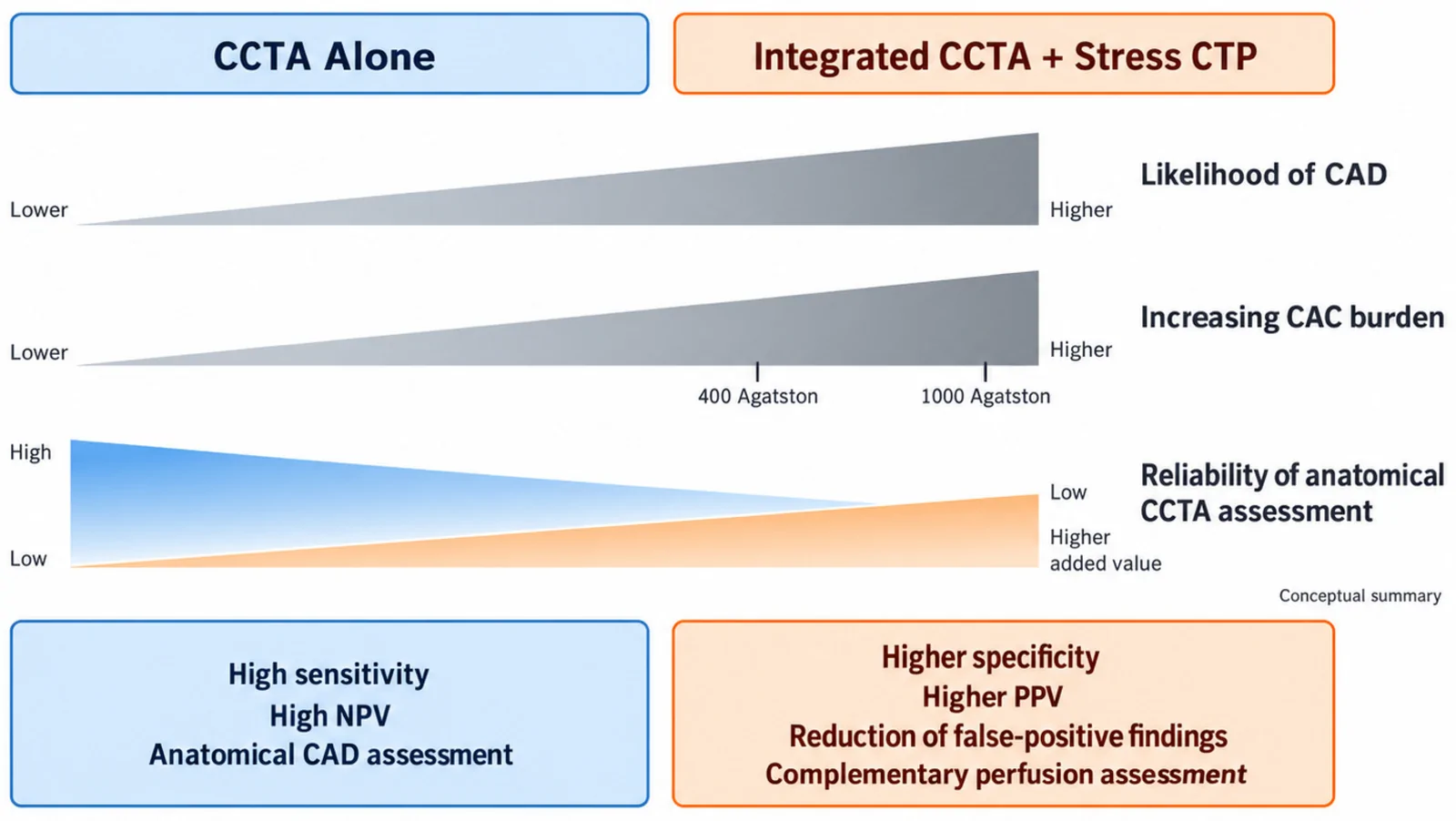
Conceptual illustration of the complementary roles of coronary CT angiography (CCTA) and stress myocardial CT perfusion (CTP) across increasing coronary artery calcium (CAC) burden. With increasing CAC, the reliability of anatomical CCTA assessment may decrease because of calcium-related artifacts, whereas stress CTP provides complementary perfusion information that may reduce false-positive anatomical classifications. **Abbreviations:** CT, computed tomography; CCTA, coronary CT angiography; CTP, CT perfusion; CAC, coronary artery calcium; CAD, coronary artery disease; NPV, negative predictive value; PPV, positive predictive value.

**Figure 3:**
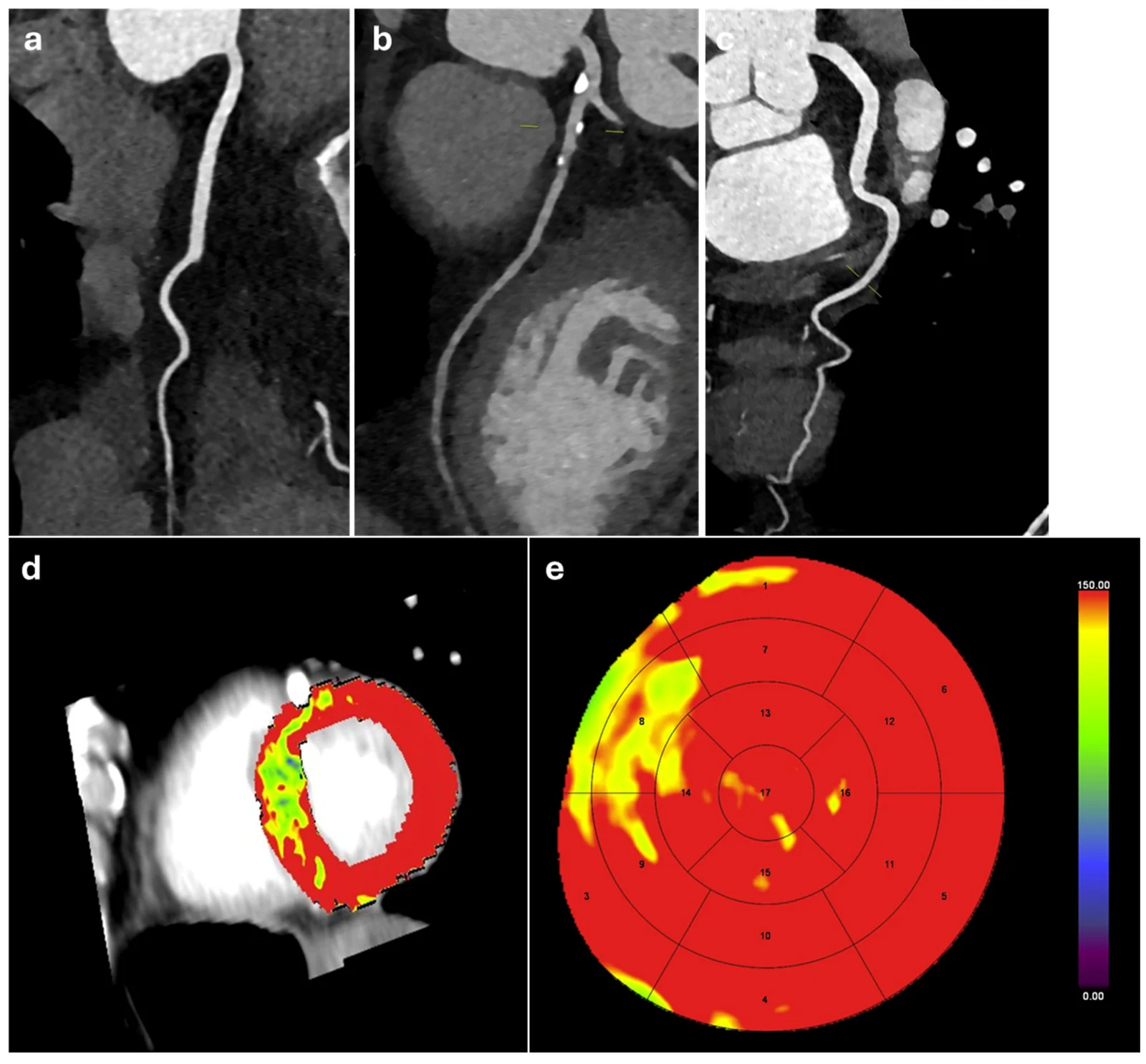
Male patient with low-to-moderate coronary calcium burden. Representative case of a patient with low-to-moderate coronary calcium burden (CAC < 400) undergoing CCTA with integrated stress myocardial CTP. (**a**–**c**) Curved multiplanar reformations of the left anterior descending artery (LAD) demonstrate moderate luminal stenosis of the mid-LAD, in the presence of limited coronary calcification. (**d**) Stress myocardial CTP short-axis image demonstrates a stress-induced perfusion defect involving the mid-to-basal segments of the interventricular septum, corresponding to the LAD territory. (**e**) Quantitative perfusion polar map confirms the presence of a stress-induced hypoperfusion in the same myocardial segments.

**Figure 4:**
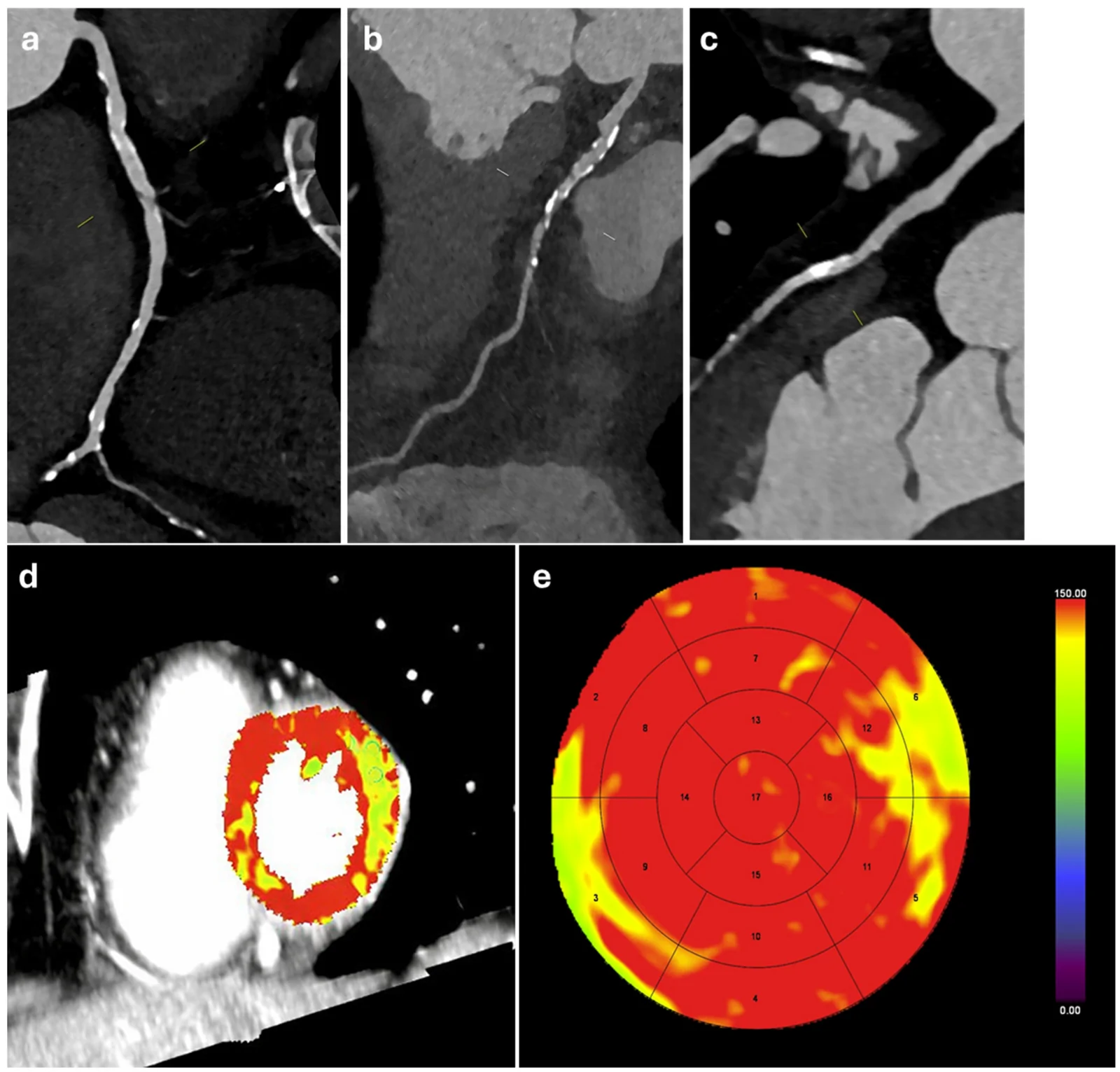
Male patient with moderate-to-high coronary calcium burden. Representative case of a patient with moderate-to-high coronary calcium burden (CAC 400–1000) undergoing coronary CT angiography (CCTA) with integrated stress myocardial CT perfusion (CTP). (**a**–**c**) Curved multiplanar reformations demonstrate moderate stenoses involving all three major coronary vessels, in the presence of diffuse coronary calcification. (**d**) Stress myocardial CTP short-axis image shows stress-induced hypoperfusion in the basal lateral wall and basal inferior interventricular septum. (**e**) Corresponding quantitative perfusion polar map confirms stress-induced hypoperfusion in the same myocardial segments.

**Figure 5:**
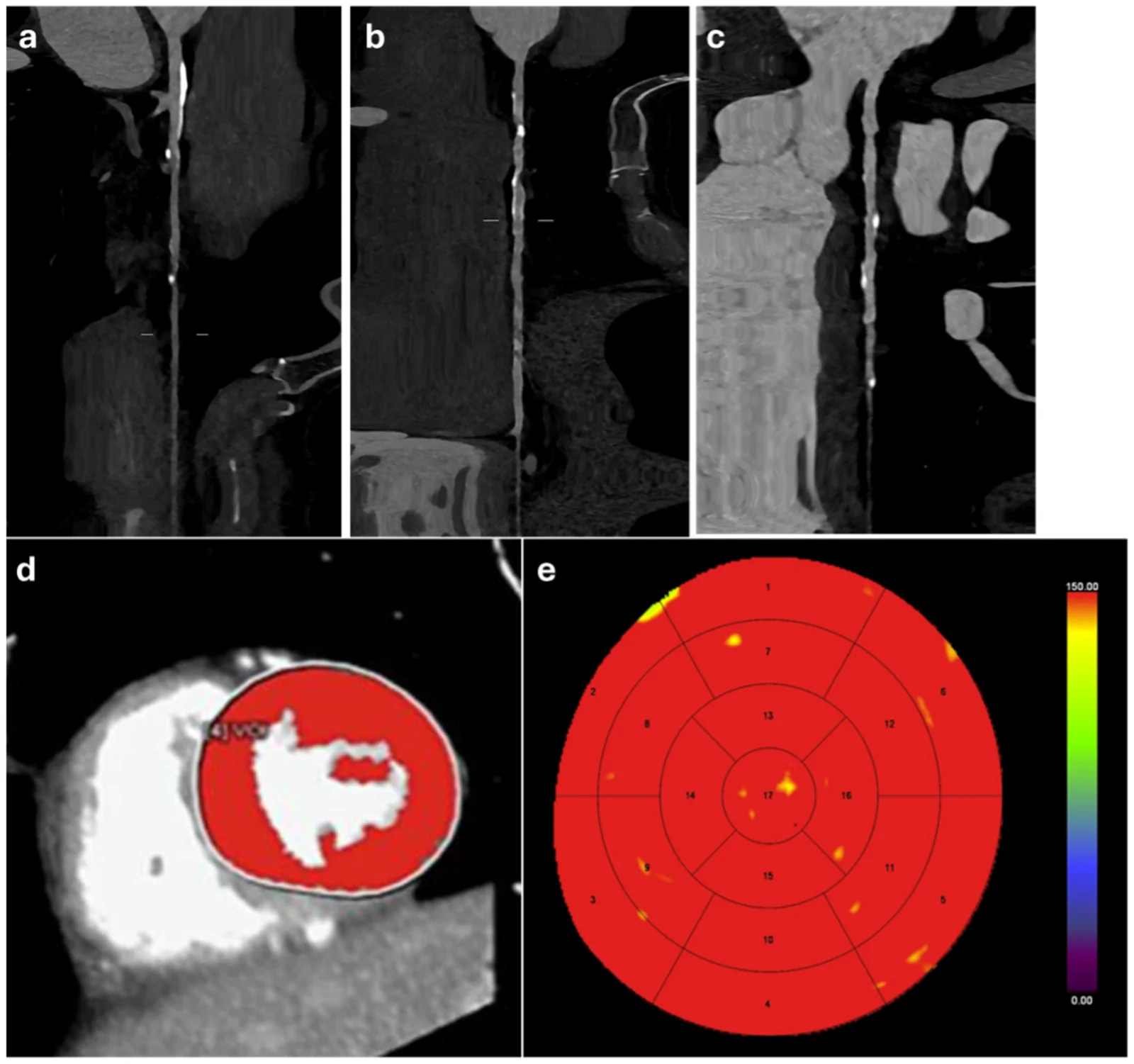
Female patient with high coronary calcium burden. Representative case of a patient with high coronary calcium burden (CAC > 1000) undergoing coronary CT angiography (CCTA) with integrated stress myocardial CT perfusion (CTP). (**a**–**c**) Curved multiplanar reformations demonstrate diffuse coronary calcification with multiple severe-appearing luminal narrowings, limiting reliable anatomical stenosis assessment. (**d**) Stress myocardial CTP short-axis image shows no evidence of stress-induced myocardial hypoperfusion. (**e**) Quantitative perfusion polar map confirms preserved myocardial perfusion across all segments.

**Table 1:** Baseline characteristics of the overall study population according to coronary artery calcium score (n = 102).

Characteristics	Total (n = 102)	CAC 0–400 (n = 38)	CAC 401–1000 (n = 43)	CAC > 1000 (n = 21)	*p*-Value
Male sex, n (%)	70 (68.6)	24 (63.2)	31 (72.1)	15 (71.4)	0.48
Age, mean ± SD (yrs)	62.5 ± 8.1	59.1 ± 7.9	64.0 ± 7.6	66.2 ± 8.3	0.01
BMI, median (Q1–Q3), kg/m^2^	27.5 (24.5–30.5)	26.7 (23.8–29.7)	27.6 (24.9–30.3)	27.9 (25.5–30.3)	0.52
Hypertension, n (%)	77 (75.5)	25 (65.8)	34 (79.1)	18 (85.7)	0.10
Type 2 diabetes, n (%)	41 (40.2)	12 (31.6)	18 (41.9)	11 (52.4)	0.22
Dyslipidemia, n (%)	81 (79.4)	30 (78.9)	34 (79.1)	17 (81.0)	0.98
Smoking (current/former), n (%)	34 (33.3)	14 (36.8)	13 (30.2)	7 (33.3)	0.82
Family history of CAD, n (%)	22 (21.6)	7 (18.4)	10 (23.3)	5 (23.8)	0.79
Presenting chest-pain category, n (%)					
– Typical angina	27 (26.5)	8 (21.1)	11 (25.6)	8 (38.1)	
– Atypical angina	53 (52.0)	22 (57.9)	23 (53.5)	8 (38.1)	
– Non-cardiac chest pain	22 (21.6)	8 (21.1)	9 (20.9)	5 (23.8)	
Additional exertional dyspnea/fatigue, n (%)	4 (3.9)	1 (2.6)	2 (4.7)	1 (4.8)	
Stress CTP hypoperfusion, n (%)	48 (47.1)	12 (31.6)	21 (48.8)	15 (71.4)	0.013

**Footnote**: Chest-pain categories were mutually exclusive and accounted for the entire study population. Exertional dyspnea/fatigue was recorded as an additional, non-mutually exclusive symptom and could therefore coexist with any of the chest-pain categories. **Abbreviations:** BMI, body mass index; CAC, coronary artery calcium; CAD, coronary artery disease; CTP, computed tomography perfusion; IQR, interquartile range; SD, standard deviation.

**Table 2:** Primary per-patient diagnostic performance of CCTA alone and integrated CCTA–stress CTP according to coronary artery calcium burden in patients with an invasive reference standard.

CAC Class	N	Reference-Positive CAD % (n/N)	Strategy	Sensitivity % (95% CI)	Specificity % (95% CI)	PPV % (95% CI)	NPV % (95% CI)	Accuracy % (95% CI)
0–400	19	57.9 (11/19)	CCTA alone	90.9 (58.7–99.8)	75.0 (34.9–96.8)	83.3 (51.6–97.9)	85.7 (42.1–99.6)	84.2 (60.4–96.6)
			CCTA + CTP	90.9 (58.7–99.8)	87.5 (47.3–99.7)	90.9 (58.7–99.8)	87.5 (47.3–99.7)	89.5 (66.9–98.7)
401–1000	30	73.3 (22/30)	CCTA alone	90.9 (70.8–98.9)	12.5 (0.3–52.7)	74.1 (53.7–88.9)	33.3 (0.8–90.6)	70.0 (50.6–85.3)
			CCTA + CTP	86.4 (65.1–97.1)	87.5 (47.3–99.7)	95.0 (75.1–99.9)	70.0 (34.8–93.3)	86.7 (69.3–96.2)
>1000	19	84.2 (16/19)	CCTA alone	87.5 (61.7–98.4)	0.0 (0.0–70.8)	82.4 (56.6–96.2)	0.0 (0.0–84.2)	73.7 (48.8–90.9)
			CCTA + CTP	87.5 (61.7–98.4)	66.7 (9.4–99.2)	93.3 (68.1–99.8)	50.0 (6.8–93.2)	84.2 (60.4–96.6)
Overall	68	72.1 (49/68)	CCTA alone	89.8 (77.8–96.6)	36.8 (16.3–61.6)	78.6 (65.6–88.4)	58.3 (27.7–84.8)	75.0 (63.0–84.7)
			CCTA + CTP	87.8 (75.2–95.4)	84.2 (60.4–96.6)	93.5 (82.1–98.6)	72.7 (49.8–89.3)	86.8 (76.4–93.8)

**Footnote:** Reference-positive CAD was defined as QCA stenosis ≥50% when FFR was unavailable and FFR ≤0.80 when performed. Thus, it should not be considered synonymous with hemodynamically significant CAD. Two-sided 95% CIs were calculated using the exact Clopper–Pearson method. CAC-stratified estimates should be interpreted cautiously because of small subgroup sizes. **Abbreviations:** CAC, coronary artery calcium; CAD, coronary artery disease; CCTA, coronary CT angiography; CTP, CT perfusion; FFR, fractional flow reserve; NPV, negative predictive value; PPV, positive predictive value; QCA, quantitative coronary angiography; CI, confidence interval.

**Table 3:** Supportive exploratory per-patient diagnostic performance using a ≥50% stenosis threshold according to CAC categories in the full study cohort.

CAC Class	N	Reference-Positive CAD % (n/N)	Strategy	Sensitivity % (95% CI)	Specificity % (95% CI)	PPV % (95% CI)	NPV % (95% CI)	Accuracy % (95% CI)
0–400	38	28.9 (11/38)	CCTA alone	90.9 (58.7–99.8)	77.8 (57.7–91.4)	62.5 (35.4–84.8)	95.5 (77.2–99.9)	81.6 (65.7–92.3)
			CCTA + CTP	90.9 (58.7–99.8)	92.6 (75.7–99.1)	83.3 (51.6–97.9)	96.2 (80.4–99.9)	92.1 (78.6–98.3)
401–1000	43	51.2 (22/43)	CCTA alone	90.9 (70.8–98.9)	61.9 (38.4–81.9)	71.4 (51.3–86.8)	86.7 (59.5–98.3)	79.1 (64.0–90.0)
			CCTA + CTP	86.4 (65.1–97.1)	90.5 (69.6–98.8)	90.5 (69.6–98.8)	86.4 (65.1–97.1)	88.4 (74.9–96.1)
>1000	21	76.2 (16/21)	CCTA alone	87.5 (61.7–98.4)	40.0 (5.3–85.3)	82.4 (56.6–96.2)	50.0 (6.8–93.2)	71.4 (47.8–88.7)
			CCTA + CTP	87.5 (61.7–98.4)	80.0 (28.4–99.5)	93.3 (68.1–99.8)	66.7 (22.3–95.7)	85.7 (63.7–97.0)
Overall	102	48.0 (49/102)	CCTA alone	89.8 (77.8–96.6)	67.9 (53.7–80.1)	72.1 (59.2–82.9)	87.8 (73.8–95.9)	78.4 (69.2–86.0)
			CCTA + CTP	87.8 (75.2–95.4)	90.6 (79.3–96.9)	89.6 (77.3–96.5)	88.9 (77.4–95.8)	89.2 (81.5–94.5)

**Footnote:** This was a supportive exploratory analysis. None of the 34 patients without ICA experienced a qualifying coronary event during complete 12-month clinical follow-up; they were therefore classified as reference-negative for this analysis. Event-free follow-up cannot exclude anatomically obstructive CAD or inducible myocardial ischemia and may consequently overestimate specificity. These estimates should not be considered equivalent to those derived from the primary invasive-reference cohort. Two-sided 95% CIs were calculated using the exact Clopper–Pearson method. CAC-stratified estimates should be interpreted cautiously because of small subgroup sizes. **Abbreviations:** CAC, coronary artery calcium; CAD, coronary artery disease; CCTA, coronary CT angiography; CTP, CT perfusion; CI, confidence interval; NPV, negative predictive value; PPV, positive predictive value.

**Table 4:** Primary per-vessel diagnostic performance using a ≥50% stenosis threshold according to CAC burden in patients with an invasive reference standard.

CAC Class	N Vessels	Reference-Positive CAD % (n/N)	Strategy	Sensitivity % (95% CI)	Specificity % (95% CI)	PPV % (95% CI)	NPV % (95% CI)	Accuracy % (95% CI)
0–400	57	36.8 (21/57)	CCTA alone	90.5 (69.6–98.8)	58.3 (40.8–74.5)	55.9 (37.9–72.8)	91.3 (72.0–98.9)	70.2 (56.6–81.6)
			CCTA + CTP	85.7 (63.7–97.0)	88.9 (73.9–96.9)	81.8 (59.7–94.8)	91.4 (76.9–98.2)	87.7 (76.3–94.9)
401–1000	90	38.9 (35/90)	CCTA alone	88.6 (73.3–96.8)	50.9 (37.1–64.6)	53.4 (39.9–66.7)	87.5 (71.0–96.5)	65.6 (54.8–75.3)
			CCTA + CTP	82.9 (66.4–93.4)	83.6 (71.2–92.2)	76.3 (59.8–88.6)	88.5 (76.6–95.6)	83.3 (74.0–90.4)
>1000	57	40.4 (23/57)	CCTA alone	87.0 (66.4–97.2)	44.1 (27.2–62.1)	51.3 (34.8–67.6)	83.3 (58.6–96.4)	61.4 (47.6–74.0)
			CCTA + CTP	78.3 (56.3–92.5)	79.4 (62.1–91.3)	72.0 (50.6–87.9)	84.4 (67.2–94.7)	78.9 (66.1–88.6)
Overall	204	38.7 (79/204)	CCTA alone	88.6 (79.5–94.7)	51.2 (42.1–60.2)	53.4 (44.5–62.2)	87.7 (77.9–94.2)	65.7 (58.7–72.2)
			CCTA + CTP	82.3 (72.1–90.0)	84.0 (76.4–89.9)	76.5 (66.0–85.0)	88.2 (81.0–93.4)	83.3 (77.5–88.2)

**Footnote:** Reference-positive CAD was defined as QCA stenosis ≥ 50% when FFR was unavailable and FFR ≤ 0.80 when performed. CAC-stratified estimates should be interpreted cautiously because of subgroup size. Per-vessel 95% CIs were estimated using patient-level cluster bootstrap resampling (2000 replications) to account for within-patient correlation among coronary vessels. FFR was available for reference classification in 36/204 vessels (17.6%) in 22 patients; the remaining 168/204 vessels (82.4%) were classified using QCA alone. **Abbreviations:** CAC, coronary artery calcium; CAD, coronary artery disease; CCTA, coronary CT angiography; CTP, CT perfusion; CI, confidence interval; NPV, negative predictive value; PPV, positive predictive value.

**Table 5:** Primary per-patient diagnostic performance using a ≥70% CCTA stenosis threshold according to CAC burden in patients with an invasive reference standard.

CAC Class	N	Reference-Positive CAD % (n/N)	Strategy	Sensitivity % (95% CI)	Specificity % (95% CI)	PPV % (95% CI)	NPV % (95% CI)	Accuracy % (95% CI)
0–400	19	57.9 (11/19)	CCTA alone	72.7 (39.0–94.0)	87.5 (47.3–99.7)	88.9 (51.8–99.7)	70.0 (34.8–93.3)	78.9 (54.4–93.9)
			CCTA + CTP	72.7 (39.0–94.0)	87.5 (47.3–99.7)	88.9 (51.8–99.7)	70.0 (34.8–93.3)	78.9 (54.4–93.9)
401–1000	30	73.3 (22/30)	CCTA alone	77.3 (54.6–92.2)	75.0 (34.9–96.8)	89.5 (66.9–98.7)	54.5 (23.4–83.3)	76.7 (57.7–90.1)
			CCTA + CTP	72.7 (49.8–89.3)	87.5 (47.3–99.7)	94.1 (71.3–99.9)	53.8 (25.1–80.8)	76.7 (57.7–90.1)
>1000	19	84.2 (16/19)	CCTA alone	81.3 (54.4–96.0)	66.7 (9.4–99.2)	92.9 (66.1–99.8)	40.0 (5.3–85.3)	78.9 (54.4–93.9)
			CCTA + CTP	81.3 (54.4–96.0)	66.7 (9.4–99.2)	92.9 (66.1–99.8)	40.0 (5.3–85.3)	78.9 (54.4–93.9)
Overall	68	72.1 (49/68)	CCTA alone	77.6 (63.4–88.2)	78.9 (54.4–93.9)	90.5 (77.4–97.3)	57.7 (36.9–76.6)	77.9 (66.2–87.1)
			CCTA + CTP	75.5 (61.1–86.7)	84.2 (60.4–96.6)	92.5 (79.6–98.4)	57.1 (37.2–75.5)	77.9 (66.2–87.1)

**Footnote:** The ≥70% threshold was applied to CCTA test positivity; the invasive reference standard remained unchanged (QCA ≥ 50% when FFR was unavailable and FFR ≤ 0.80 when performed). Two-sided 95% CIs were calculated using the exact Clopper–Pearson method. **Abbreviations:** CAC, coronary artery calcium; CAD, coronary artery disease; CCTA, coronary CT angiography; CTP, CT perfusion; NPV, negative predictive value; PPV, positive predictive value.

**Table 6:** Primary per-vessel diagnostic performance using a ≥70% CCTA stenosis threshold according to CAC burden in patients with an invasive reference standard.

CAC Class	N Vessels	Reference-Positive CAD % (n/N)	Strategy	Sensitivity % (95% CI)	Specificity % (95% CI)	PPV % (95% CI)	NPV % (95% CI)	Accuracy % (95% CI)
0–400	57	36.8 (21/57)	CCTA alone	76.2 (52.8–91.8)	83.3 (67.2–93.6)	72.7 (49.8–89.3)	85.7 (69.7–95.2)	80.7 (68.1–90.0)
			CCTA + CTP	71.4 (47.8–88.7)	91.7 (77.5–98.2)	83.3 (58.6–96.4)	84.6 (69.5–94.1)	84.2 (72.1–92.5)
401–1000	90	38.9 (35/90)	CCTA alone	77.1 (59.9–89.6)	76.4 (63.0–86.8)	67.5 (50.9–81.4)	84.0 (70.9–92.8)	76.7 (66.6–84.9)
			CCTA + CTP	71.4 (53.7–85.4)	90.9 (80.0–97.0)	83.3 (65.3–94.4)	83.3 (71.5–91.7)	83.3 (74.0–90.4)
>1000	57	40.4 (23/57)	CCTA alone	78.3 (56.3–92.5)	61.8 (43.6–77.8)	58.1 (39.1–75.5)	80.8 (60.6–93.4)	68.4 (54.8–80.1)
			CCTA + CTP	73.9 (51.6–89.8)	82.4 (65.5–93.2)	73.9 (51.6–89.8)	82.4 (65.5–93.2)	78.9 (66.1–88.6)
Overall	204	38.7 (79/204)	CCTA alone	77.2 (66.4–85.9)	74.4 (65.8–81.8)	65.6 (55.0–75.1)	83.8 (75.6–90.1)	75.5 (69.0–81.2)
			CCTA + CTP	72.2 (60.9–81.7)	88.8 (81.9–93.7)	80.3 (69.1–88.8)	83.5 (76.0–89.3)	82.4 (76.4–87.3)

**Footnote:** The ≥70% threshold was applied to CCTA test positivity; the invasive reference standard remained unchanged. CAC-stratified estimates should be interpreted cautiously because of subgroup size. Per-vessel 95% CIs were estimated using patient-level cluster bootstrap resampling (2000 replications) to account for within-patient correlation among coronary vessels. FFR was available for reference classification in 36/204 vessels (17.6%); the remaining 168/204 vessels (82.4%) were classified using QCA alone. **Abbreviations:** CAC, coronary artery calcium; CAD, coronary artery disease; CCTA, coronary CT angiography; CTP, CT perfusion; NPV, negative predictive value; PPV, positive predictive value.

**Table 7:** Supportive exploratory per-patient diagnostic performance using a ≥70% CCTA stenosis threshold according to CAC burden in the full study cohort.

CAC Class	N	Reference-Positive CAD % (n/N)	Strategy	Sensitivity % (95% CI)	Specificity % (95% CI)	PPV % (95% CI)	NPV % (95% CI)	Accuracy % (95% CI)
0–400	38	28.9 (11/38)	CCTA alone	72.7 (39.0–94.0)	92.6 (75.7–99.1)	80.0 (44.4–97.5)	89.3 (71.8–97.7)	86.8 (71.9–95.6)
			CCTA + CTP	72.7 (39.0–94.0)	96.3 (81.0–99.9)	88.9 (51.8–99.7)	89.7 (72.6–97.8)	89.5 (75.2–97.1)
401–1000	43	51.2 (22/43)	CCTA alone	77.3 (54.6–92.2)	85.7 (63.7–97.0)	85.0 (62.1–96.8)	78.3 (56.3–92.5)	81.4 (66.6–91.6)
			CCTA + CTP	72.7 (49.8–89.3)	95.2 (76.2–99.9)	94.1 (71.3–99.9)	76.9 (56.4–91.0)	83.7 (69.3–93.2)
>1000	21	76.2 (16/21)	CCTA alone	81.3 (54.4–96.0)	60.0 (14.7–94.7)	86.7 (59.5–98.3)	50.0 (11.8–88.2)	76.2 (52.8–91.8)
			CCTA + CTP	81.3 (54.4–96.0)	80.0 (28.4–99.5)	92.9 (66.1–99.8)	57.1 (18.4–90.1)	81.0 (58.1–94.6)
Overall	102	48.0 (49/102)	CCTA alone	77.6 (63.4–88.2)	86.8 (74.7–94.5)	84.4 (70.5–93.5)	80.7 (68.1–90.0)	82.4 (73.6–89.2)
			CCTA + CTP	75.5 (61.1–86.7)	94.3 (84.3–98.8)	92.5 (79.6–98.4)	80.6 (68.6–89.6)	85.3 (76.9–91.5)

**Footnote:** This was a supportive exploratory analysis. None of the 34 patients without ICA experienced a qualifying coronary event during the 12-month follow-up; therefore, all 34 were classified as reference-negative for this analysis. Event-free follow-up cannot exclude obstructive CAD or inducible myocardial ischemia and may therefore overestimate specificity. **Abbreviations:** CAC, coronary artery calcium; CAD, coronary artery disease; CCTA, coronary CT angiography; CTP, CT perfusion; NPV, negative predictive value; PPV, positive predictive value.

## Data Availability

The data presented in this study are available on request from the corresponding author.
